# Chronic sleep fragmentation shares similar pathogenesis with neurodegenerative diseases: Endosome‐autophagosome‐lysosome pathway dysfunction and microglia‐mediated neuroinflammation

**DOI:** 10.1111/cns.13218

**Published:** 2019-09-24

**Authors:** Yi Xie, Li Ba, Min Wang, Sai‐Yue Deng, Si‐Miao Chen, Li‐Fang Huang, Min Zhang, Wei Wang, Feng‐Fei Ding

**Affiliations:** ^1^ Department of Neurology Tongji Hospital Tongji Medical College Huazhong University of Science and Technology Wuhan China; ^2^ Key Laboratory of Neurological Diseases of Chinese Ministry of Education The School of Basic Medicine Tongji Medical College Huazhong University of Science and Technology Wuhan China

**Keywords:** Alzheimer's disease, amyloid‐β, autophagy, lysosome, microglia, sleep fragmentation

## Abstract

**Aims:**

Insufficient sleep has been found to result in varying degrees of cognitive impairment and emotional changes. Sleep was reported probably responsible for cleaning metabolic wastes in brain by increasing extracellular bulk flow. Herein, we propose that chronic sleep insufficiency in young adult wild‐type mice is also linked with dysfunction of intracellular protein degradation pathways and microglia‐mediated neuroinflammation, which are potentially important mechanisms in the initiation of neurodegeneration.

**Methods:**

We applied the chronic sleep fragmentation (CSF) model to induce chronic sleep insufficiency in wild‐type mice. After 2 months of CSF, cognitive function, amyloid‐β accumulation, dysfunction of endosome‐autophagosome‐lysosome pathway, and microglia activation were evaluated.

**Results:**

Following CSF, impairment of spatial learning and memory, and aggravated anxiety‐like behavior in mice were identified by behavioral experiments. Increased intracellular amyloid‐β accumulation was observed in cortex and hippocampus. Mechanistically, CSF could significantly enhance the expression of Rab5 (early endosome marker), Rab7 (late endosome marker), as well as LC3B (autophagosome marker), and autophagy‐positive regulatory factors in brain detected by immunofluorescent staining and Western blot. In addition, activation of microglia was evident by enhanced CD68, CD16/32, and CD206 levels after CSF treatment.

**Conclusions:**

Chronic sleep fragmentation could initiate pathogenetic processes similar to the early stage of neurodegeneration, including dysfunction of endosome‐autophagosome‐lysosome pathway and microglia‐mediated neuroinflammation. Our findings further strengthen the link between chronic sleep insufficiency and the initiation of neurodegeneration even if lack of genetic predisposition.

## 1 INTRODUCTION

Lack of sleep has already become a pervasive trend in many populations in our society, mostly due to electronic devices use, excessive workload, and life stress. People, especially the younger population, are usually ignorant of the importance of sleep and holding inappropriate sleep habits for long. Adequate sleep has been believed partially participate in waste clearance in the brain and memory consolidation.^1^, ^2^ Given substantial evidence, chronic sleep insufficiency is associated with cognitive decline and psychological problems ranging from mood changes to psychotic symptoms.^3^, ^4^ According to the most recent meta‐analysis, individuals with sleep disturbance were 1.55 (95% confidence intervals: 1.25‐1.93) and 3.78 (95% confidence intervals: 2.27‐6.30) times as likely to have Alzheimer's disease (AD) and preclinical AD than individuals with normal sleep.^5^ Clinical observations suggested that neurodegenerative diseases such as AD and Parkinson disease, among others, usually exhibited sleep disturbance in the preclinical and early stages of the disease process.^6^ Sleep disruption that was untreated could in turn accelerate neurodegenerative disease progression.^7^ Briefly, sleep insufficiency could potentially aggravate neurodegeneration and vice versa.

Alzheimer's disease, the most common type of neurodegenerative diseases, is generally characterized by memory loss, spatial learning disorder, and behavioral changes.^8^ Only a small proportion of AD cases are familial, which is caused by gene mutations in the amyloid precursor protein (APP), presenilin 1 (PS1), and presenilin 2 (PS2) leading to increased production of Aβ.^9^, ^10^ However, in the far more common sporadic cases, Aβ generation is normal, but its clearance is impaired.^11^ It was recently reported that sleep was an important physiological process, during which extracellular metabolic wastes such as β‐amyloid protein were cleared via paravascular pathway.^1^, ^12^, ^13^ Meanwhile, this paravascular cleaning processes were found to be significantly suppressed by sleep deprivation treatment and in AD mouse models.^14^, ^15^, ^16^ Clinical studies have confirmed that neurodegenerative pathogenesis was initiated more than 20 years prior to positive detection of extracellular mis‐folded protein deposits and symptoms of clinically evident cognitive decline.^17^ The preclinical stage could thus be much more important than late stages for the development of effective interventions for neurodegenerative diseases in clinical practice.

Intracellular and extracellular amyloid‐β (Aβ) protein accumulation is a typical pathological change that plays a pivotal role in the evolution of AD.^18^, ^19^ In normal conditions, APP, the precursor of Aβ, is internalized into cells by endocytosis and then fused with either the lysosome or autophagosome for lysosomal degradation.^19^ The intracellular integrated protein degradation pathway involving endosomes, autophagosomes, and lysosomes is termed the endosome‐autophagosome‐lysosome (EAL) pathway.^20^ EAL pathway dysfunction is characterized by progressive accumulation of autophagic vacuoles (AVs) and enlargement of endosomes, which can also be seen early in AD neuropathogenesis.^20^, ^21^ Our recent data reported altered expression of endosome, autophagosome, and lysosome markers in AD transgenic mice,^20^ which supported the hypothesis that EAL pathway dysregulation could be a causative mechanism for AD initiation. Furthermore, microglia activation due to accumulation of Aβ has also been believed to participate in the pathogenesis of AD.^22^, ^23^, ^24^ Microglia activation could induce neuronal damage by producing proinflammatory cytokines, chemokines, complement proteins, and upon strong activation may release toxic‐free radicals.^25^, ^26^ Neuroinflammation was proposed closely linked to and may even precede the development of other neuropathological characteristics of AD.^27^

Previous study indicated that sleep deprivation treatment accelerates neurodegeneration in APP/PS‐mutated mice.^28^ However, in the current study, we provided evidence that chronic sleep fragmentation (CSF) could induce similar pathogenesis of neurodegeneration in young adult wild‐type mice, involving dysfunction of intracellular protein degradation as well as microglia activation. Our data would highlight the potential risk of initiating neurodegeneration by chronic sleep insufficiency, even if in young adults without genetic predisposition of neurodegenerative diseases.

## 2 MATERIALS AND METHODS

### 2.1 Animals and model

All animal experiments were approved by the Institutional Animal Care and Use Committee of Tongji Hospital, Tongji Medical College, Huazhong University of Science and Technology (Permit Number: TJ‐A20171204). Wild‐type C57BL/6J mice were obtained from Hubei Research Center for Laboratory Animals, Hubei, China. Adult male mice (20‐28 g; 10‐12 weeks old) were randomly assigned into the CSF group and the control group, all of which were maintained in a 12‐hour light‐dark cycle (8:00 am‐8:00 pm light‐ON, 8:00 pm‐8:00 am light‐OFF), with free access to food and water.

**Figure 1:**
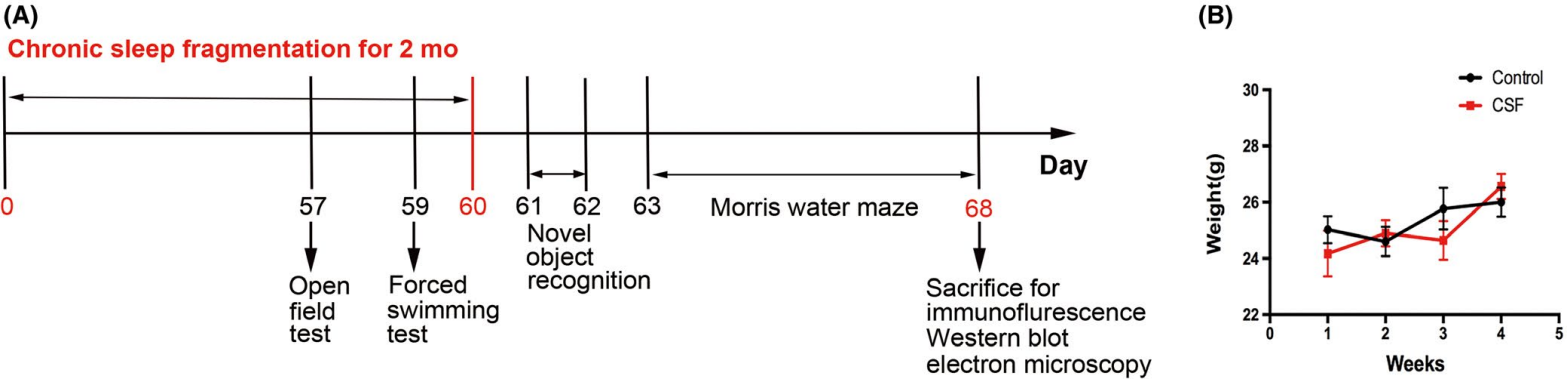
The schematic figure of experimental design procedure. A, The experimental design procedure that indicates the timing of CSF model, behavioral tests, immunofluorescence, electron microscopy, and Western blot. B, Body weight curves of the CSF and control mice during the first month after the CSF model were established

The model of sleep fragmentation was induced using methods validated by Sinton et al^29^ and modified by Sigrid et al.^30^ Briefly, we used an orbital rotor (Shiping) with a speed of 55 Hz and a repeated cycle of 10‐second on, 110‐second off, during light‐ON phase (8:00 am‐8:00 pm) continuously for 2 months. Prior to the study, we ensured that all mice were able to groom, eat, and drink during orbital rotor movement of the platform, yet would arouse from sleep upon orbital rotor movement. The experimental design procedure is illustrated in order of time (Figure 1A). No difference was observed in the weight increase in mice between two groups during the first month of CSF (Figure 1B).

### 2.2 Morris water maze (MWM) test

The Morris water maze test is widely used to assess spatial learning and memory performance in rodents.^31^ The apparatus consisted of a circular tank (150 cm in diameter and 40 cm deep) filled with warm water (20‐23°C). Four different pictures were hung on the curtain surrounding the tank in four quadrant directions to function as permanent distal cues. The water was made to appear opaque by the addition of powdered milk. A platform (10 cm in diameter) was located in the middle of the southwest quadrant. Mice were subjected to four consecutive trials between 8:00 and 12:00 am each day over a 5‐days training period. Each mouse was released from four different positions around the perimeter of the tank (north, northwest, east, and southeast) in four trials in the same order.^32^ In each trial, every mouse was allowed to swim until it found the platform (for a maximum of 60 seconds). If the platform was not found in 60 seconds, the mouse was guided to the platform and remained there for 15 seconds. The escape latency to find the hidden platform was automatically recorded using a video tracking system (XR). On the sixth day after 5‐training days, a probe test was conducted. The platform was removed while each mouse was released from the northeast quadrant and was allowed to swim for 60 seconds. Memory retention was measured by quantifying the time spent in the target quadrant (southwest) and the number of times the mouse crossed the previous platform location.

### 2.3 Novel object recognition (NOR) test

Mice were subjected to the novel object recognition test to assess their object recognition and short‐time working memory.^33^ During the familiar phase, mice were placed in the box (length 30 cm, width 28 cm, height 35 cm) in sequence which contained two copies of objects (A1 and A2 both in red, cylindrical wood), and allowed to explore freely (10 minutes per trial). After a 1‐hour delay, the test trial was conducted. The mice were returned to the box in which one of the original objects was replaced by a novel object in green, cubic plastic with similar size. (“novel”), while the other object remained unchanged (“familiar”) (5 minutes per trial). Exploration of the object was defined by sniffing, licking, chewing, or moving vibrissae while directing the nose toward and less than 1 cm from the object. The number of episodes and time spent in exploration of each object by each animal were recorded using a video camera (XR). The discrimination index between the two objects was calculated as DI = (TN − TF)/(TN + TF) (TN = time spent exploring the “novel” object, TF = time spent exploring the “familiar” object). In the familiar phase, the total exploration time (Te) and the exploration time for A1 and A2 objects were also recorded.

### 2.4 Open field test (OFT)

The open field test was used to assess the locomotor activity and anxiety‐like behavior^34^, ^35^, ^36^ of the control and CSF groups. The apparatus consisted of a tank (length 30 cm, width 28 cm, height 35 cm) with a video camera (XR) fixed at the top of the tank (XR). During the tests, each animal was placed into the center of the tank and allowed to explore freely for 5 minutes. The tank was cleaned with 75% ethanol after every test to avoid the leftover effects by previous mice. The total distances and average speed during the 5‐minute period were recorded automatically as the index of locomotor activity. The distance to the center zone and inner toroid time was used as exploratory behavior to assess the level of anxiety.

### 2.5 Forced swimming test (FST)

The forced swimming test was performed to measure the depression‐like behavior of the control and CSF groups.^37^ Mice were individually forced to swim in an open cylindrical vessel, which contained water 15 cm deep at a temperature of 20‐23°C, so that they could not escape or touch the bottom. Each mouse was gently placed in the cylinder, and the total duration of floating was recorded during a 6‐minute period. The immobility time was measured during the last 4 minutes of the test. Each mouse was judged to be immobile when it stopped struggling and maintained motionless floating in the water, making only those movements necessary to keep its head above water.

### 2.6 Tissue preparation and immunofluorescent staining

Mice were anesthetized and then perfused with 20 mL of 4% paraformaldehyde. After perfusion, brains were removed, postfixed in 4% paraformaldehyde (4℃), and then embedded in paraffin. Coronal slices with thickness of 4 μm were prepared and mounted using a rotary microtome (RM2016, Leica). For immunofluorescence staining, the paraffin‐embedded sections were deparaffinized in xylene and rehydrated in graded ethanol solutions, respectively. Then, the slices were placed in a water bath at 96°C for 20 minutes for antigen retrieval. After washed twice with PBS, the sections were blocked in 10% bovine serum albumin for 1 hour at room temperature and then incubated with primary antibodies overnight at 4°C. The following primary antibodies were used, rabbit anti‐Rab5 (ab18211, Abcam), mouse anti‐Rab7 (ab18211), rabbit anti‐LC3B (3868S, Cell Signaling Technology), rabbit anti‐Lamp1 (ab24170, Abcam), goat anti‐Beclin 1 (sc10086), rabbit anti‐UVRAG (ab24170, Millipore), rabbit anti‐Amyloidβ1‐42 (ab201060, Abcam), rat anti‐CD68 (MCA1957GA, Bio‐Rad), rabbit anti‐Iba1 (019‐19741), rat anti‐CD16/32(019‐19741, BD Biosciences), and goat anti‐CD206 (AF2535, R&D). After conjugation with the primary antibodies, the sections were rinsed in PBS and then incubated with the corresponding secondary antibodies (Jackson ImmunoResearch Laboratories) for 1 hour at room temperature. Finally, sections were observed blindly under an Olympus BX51 fluorescence microscope (Olympus) or laser scanning confocal microscope (Olympus, FV500). The number of positively labeled cells per square millimeter (cells/mm^2^) in five fields was quantified by a blinded investigator using ImageJ software (National Institutes of Health).

### 2.7 Electron microscopy

Mice were anesthetized and perfused with 4% paraformaldehyde/2.5% glutaraldehyde buffered with PBS. The prefrontal cortex was removed, cut into 1‐mm‐thick sections, and then immersed in 2.5% glutaraldehyde overnight at 4°C. The samples were postfixed in 1% OsO4 for 2 hours at 4°C in the dark, block‐stained in 1% uranyl acetate for 1 hour, dehydrated in a graded series of aqueous alcohol solutions, and embedded in epoxy resin. Slices with thickness of 70 nm were prepared and mounted using an ultramicrotome (UC7, Leica). Slices were observed using a CM‐120 transmission electron microscope (FEI UK Ltd.). Fifteen fields per sample were randomly captured. The number and size of lysosomes between the CSF and control groups were compared.

### 2.8 Western blot

For Western blotting, the cortex and hippocampus of the mice were quickly removed and homogenized in RIPA lysis buffer with protease inhibitor cocktail (Beyotime). After centrifugation at 10 600 *g* at 4°C for 15 minutes, the supernatants were collected, and the protein concentration was determined using a BCA Kit (Beyotime). Then, the proteins were mixed with loading buffer and boiled for 10 minutes. Samples containing 30 μg total protein were loaded on 10% SDS‐PAGE gels. After electrophoresis, the proteins were transferred to the nitrocellulose membrane (0.45 μm, Millipore). The nonspecific binding was blocked by 5% nonfat milk in Tris‐buffered saline (TBS) containing 0.1% Tween‐20 for 1 hour at room temperature. The membrane was then incubated with the following primary antibodies: rabbit anti‐Rab5 (ab18211, Abcam), mouse anti‐Rab7 (ab18211, Sigma), rabbit anti‐LC3B (L7543, sigma), rabbit anti‐Lamp1 (ab24170, Abcam), mouse anti‐Beclin 1 (612278, BD Biosciences), rabbit anti‐UVRAG (AB2960, Millipore), rat anti‐CD16/32 (A01408‐1, Boster), goat anti‐CD206 (AF2535, R&D system), rabbit anti‐iNOS (A0312, Abclonal), and rabbit anti‐β‐actin (GB11001, Servicebio). Thereafter, the membranes were incubated with the appropriate HRP‐conjugated secondary antibodies (Jackson ImmunoResearch) for 1 hour at room temperature. Finally, the blots were visualized using a Bio‐Rad ChemiDoc XRS+ imaging system with enhanced chemiluminescence kits (Advansta ECL). The bands were analyzed blindly using ImageJ software to obtain the optical densities (OD) of the signals. The value was expressed as the ratio of OD of the tested proteins to OD of β‐actin.

### 2.9 Statistical analysis

All measurements were performed by researchers blinded to each group and condition. Data were expressed as the means ± SEM. Differences between the two groups of mice in the escape latency of the MWM test were determined by two‐way ANOVA with repeated measures followed by Bonferroni posttests. Other comparisons between the CSF and control groups were determined by *t* tests. Data were analyzed using GraphPad Prism 6.0 (GraphPad Software, Inc). Differences were considered significant if *P* < .05 in all tests.

## 3 RESULTS

### 3.1 CSF caused cognitive decline and aggravated anxiety

**Figure 2:**
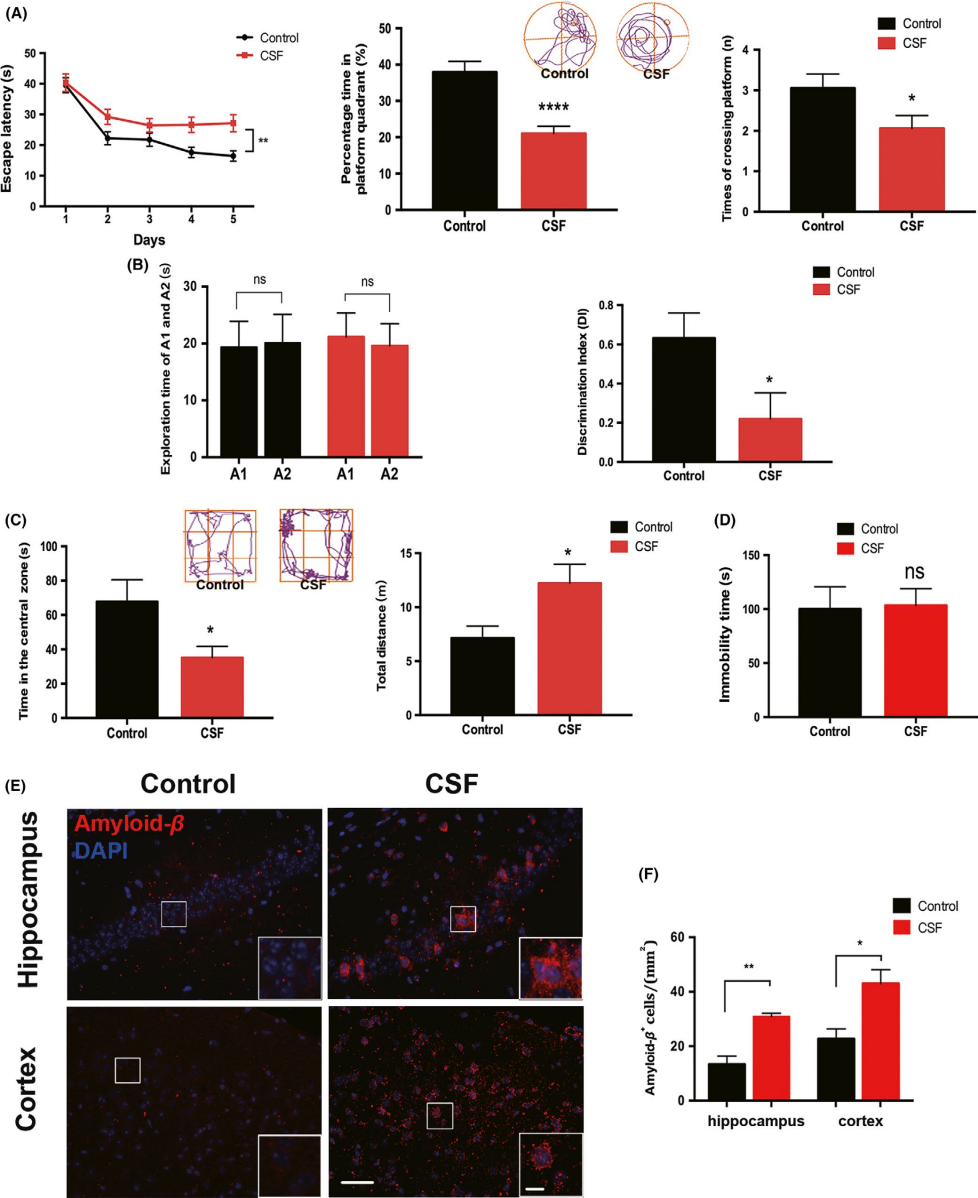
CSF aggravated cognitive impairment and increased intracellular Aβ accumulation. A, Left, escape latency in acquisition phase; middle and right, percentage time in the platform quadrant and times of crossing the platform in probe trial. Representative tracings of two groups were shown in the upper panel. n = 10 per group, *P < .05, **P < .01, ****P < .0001. B, Left, exploration time of A1 and A2, respectively, by mice in familiar phase; right, discrimination index (DI) in the test phase. n = 10 per group, n.s. indicates no significant changes between different groups, *P < .05. C, Left, time in the central zone; right, total distance travelled by the mice. Representative tracings of two groups were shown in the upper panel. n = 10 per group, *P < .05. D, Immobility time during the last 4 min of the test. n = 10 per group, n.s. indicates no significant changes between different groups. E, Representative immunofluorescence images of intracellular Aβ accumulation in the cortex and hippocampus of CSF and control mice. Scale bar = 40 μm. Local enlarged images were presented in the white squares. Scale bar = 10 μm. F, Quantitative analysis of the number of Aβ^+^ cells was shown in the histogram, n = 5 per group, *P < .05, **P < .01

To assess the effects of CSF on learning and memory, we performed behavioral tests of MWM and NOR. The MWM test showed that CSF group had longer escape latency to find the platform through five training days vs the control group. Moreover, in the probe test, CSF mice spent significantly less time in the targeted quadrant and exhibited a fewer number of crossings in the area where the platform was previously located (Figure 2A). These results suggested significant deficits in spatial learning and memory after CSF. In the NOR test, no difference was found in the exploration time for the object A1 and the object A2 in the familiar phase between the two groups. However, in the test phase, the Discrimination Index (DI) of the CSF group was significantly decreased compared with that of the control group (Figure 2B), which indicates deficits in object recognition.

To further explore the influence of CSF on behavior changes, the OFT and FST were conducted. Interestingly, in the OFT, we found that the CSF group displayed longer total distance moved in the tank, indicating an increase in spontaneous activities. Additionally, CSF mice spent less time in the central zone during the observed 5 minutes (Figure 2C), which demonstrated that sleep disturbance could aggravate anxiety‐like behavior. Nevertheless, no evident depression‐like behavior was observed in this model, considering nonsignificant difference in the immobility time between two groups subjected to the FST (Figure 2D).

### 3.2 CSF significantly increased intracellular β‐amyloid(Aβ) accumulation

Using immunofluorescence staining, we observed elevated number of Aβ^+^ cells in the cortex and hippocampus of CSF mice compared with control mice (Figure 2E,F). The detected Aβ was primarily distributed within the cells, while extracellular Aβ deposits were not detected after 2‐month CSF.

### 3.3 CSF caused accumulation of endosomes and lysosomes

**Figure 3:**
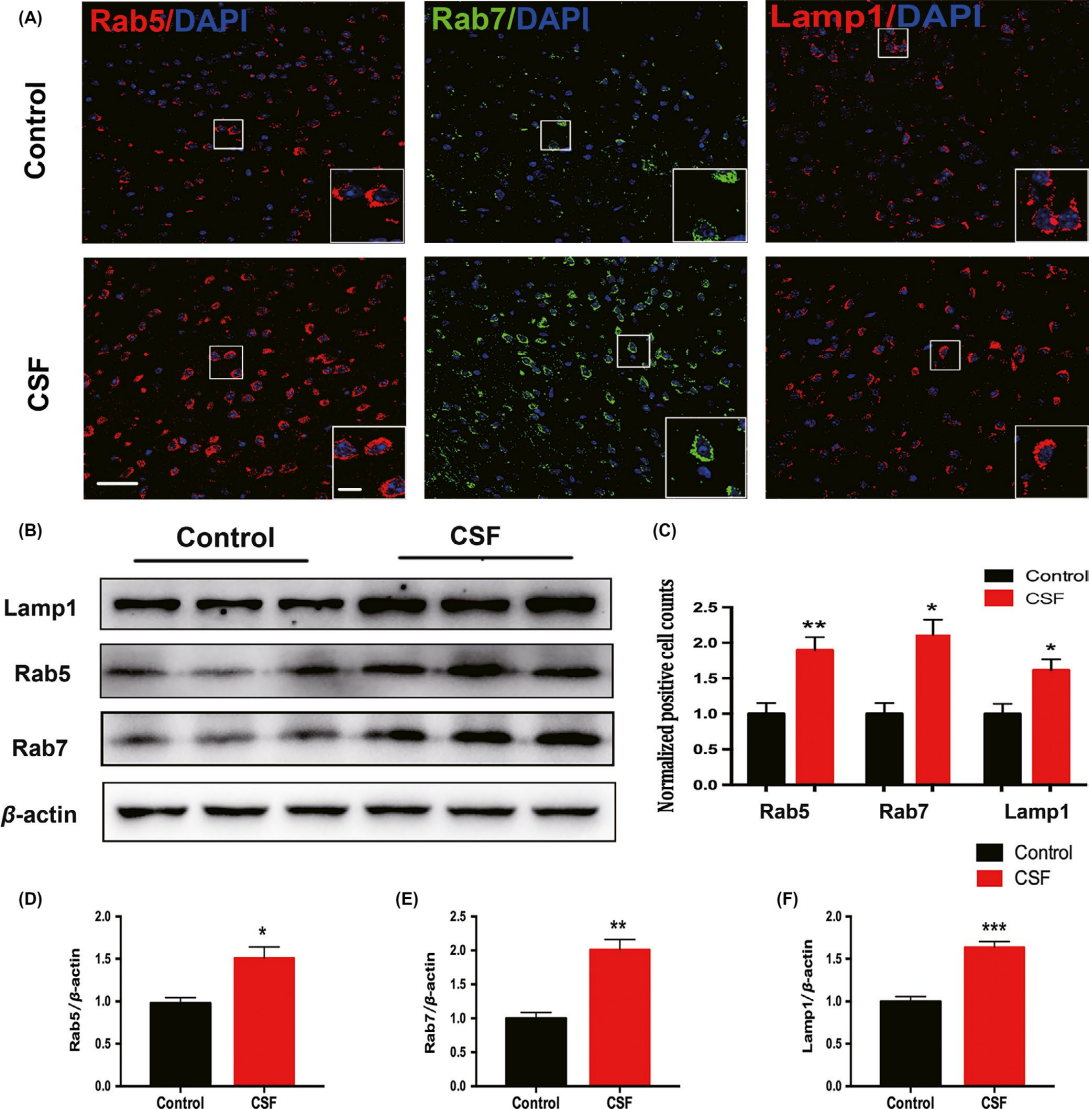
Intracellular endosomes and lysosomes in the cortex were dysregulated after CSF. A, Representative confocal images of coronal sections labeled by Rab5, Rab7, and Lamp1 staining in the cortex of CSF and control mice. Scale bar = 40 μm. Local enlarged images were presented in the white squares. Scale bar = 10 μm. B, Representative Western blots showing the changes in the expression of Rab5, Rab7, and Lamp1 in the cortex of CSF and control mice. C, Histograms showed the quantitative density of cells immunoreactive for Rab5, Rab7, and Lamp1. n = 5 per group, *P < .05, **P < .01. D‐F, Quantitative analysis of the Rab5, Rab7, and Lamp1 protein levels was performed. Protein expression levels were normalized to the level of β‐actin. n = 5 per group, *P < .05, **P < .01, ***P < .001

It has been found that APP is first internalized by endocytosis and sorted in early endosomes, then delivered to late endosomes, both of which are enriched in APP secretases.^38^ The late endosomes fuse with either lysosomes or autophagosomes for Aβ degradation.^19^, ^20^ Herein, we measured the expression of the early endosome marker Rab5 and the late endosome marker Rab7, which were both involved in the trafficking, catabolism, and elimination of APP to generate Aβ.^39^, ^40^, ^41^ The CSF‐induced increase in the numbers of Rab5^+^ and Rab7^+^ cells in the cortex was evident by immunofluorescence staining (Figure 3A,C). Consistently, Western blot results showed similar alterations in Rab5 and Rab7 expression at protein level in this CSF model (Figure 3B,D,E).

**Figure 4:**
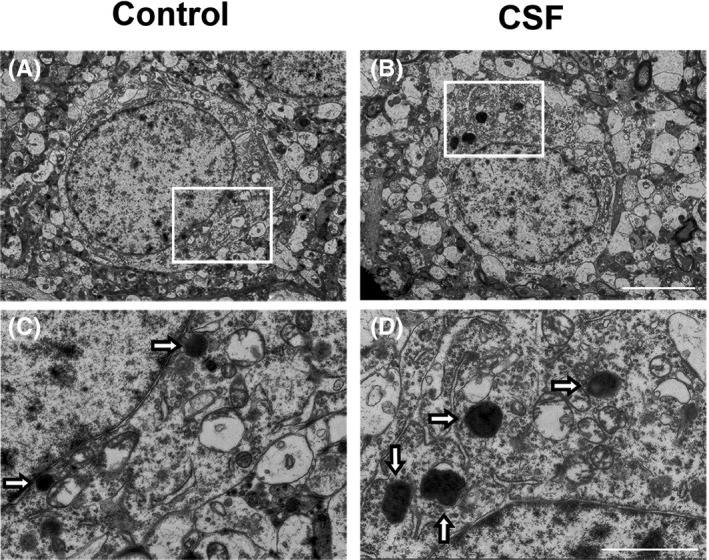
CSF caused enlargement and increase in intracellular lysosomes in the cortex detected by electron microscopy. A‐B, Representative electron photomicrographs of neurons from mice in the CSF and control groups. Scale bar = 5 μm. C‐D, Higher magnification images of the rectangle area in (A‐B), hollow arrows marked intracellular lysosomes. Scale bar = 2 μm

We next evaluated the lysosome marker Lamp1. The density of Lamp1^+^ cells and its protein expression in the cortex were both enhanced by CSF, which indicates the accumulation and dysregulation of cellular lysosomes (Figure 3A‐C,F). Based on the above observations, we further used the electron microscopy to determine the ultrastructural changes in lysosomes in CSF mice. As shown in these electron microscopy images, we found more abundant and enlarged lysosomes in the CSF cortex vs the control cortex (Figure 4).

### 3.4 CSF induced disorder of the autophagy process

**Figure 5:**
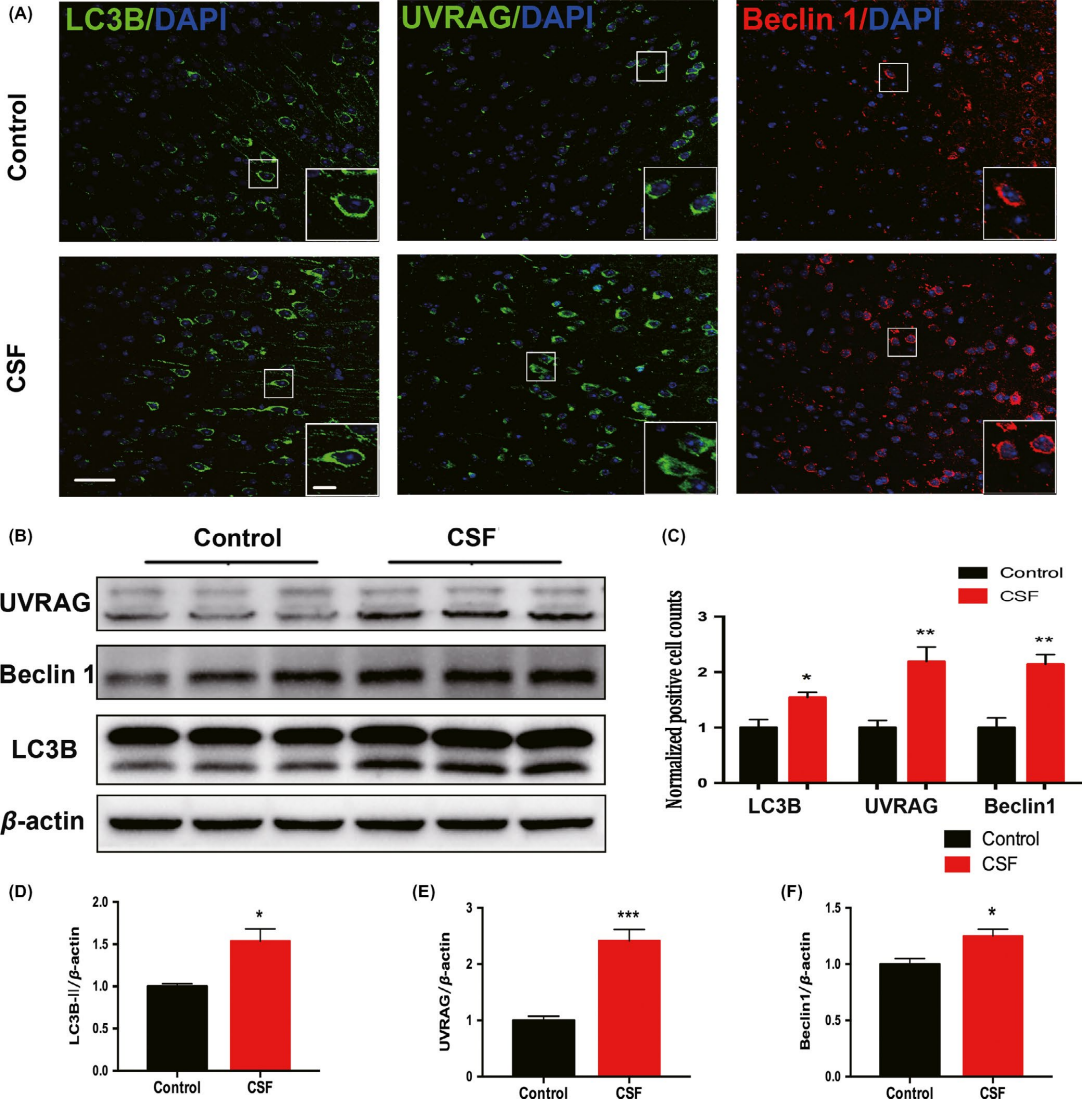
Intracellular autophagy process in the cortex was disordered after CSF. A, Representative confocal images of coronal sections labeled by LC3B, Beclin 1, and UVRAG staining in the cortex of CSF and control mice. Scale bar = 40 μm. Local enlarged images were presented in the white squares. Scale bar = 10 μm. B, Representative Western blots showing the changes in the expression of LC3B‐II, Beclin 1, and UVRAG in the cortex of CSF and control mice. C, Histograms showed the quantitative density of cells immunoreactive for LC3B, Beclin1, and UVRAG. n = 5 per group,*P < .05, **P < .01. D‐F, Quantitative analysis of the LC3B‐II, Beclin1, and UVRAG protein levels was performed. The protein expression levels were normalized to the level of β‐actin. n = 5 per group, *P < .05, **P < .01

Autophagy is responsible for degrading aggregated proteins and damaged organelles within autophagosomes or by fusion with the lysosomes.^42^ Overactive autophagy and accumulated AVs are characteristics of the early stage of neurodegeneration.^20^, ^21^ Our data showed a significant increase in the number of LC3B^+^ (autophagosome marker) cells in the cortex of CSF mice (Figure 5A,C). In agreement with the immunofluorescence results, the LC3B‐II protein level was also elevated after CSF (Figure 5B,D).

To further explore the changes in autophagy, we examined the expression of two autophagy‐positive regulatory factors (Beclin1 and UVRAG) in the cortex by immunofluorescence staining and Western blot. As shown in the figures, the expression of Beclin 1 and UVRAG both increased significantly in the CSF group compared with the control group (Figure 5A‐C,E,F).

### 3.5 CSF induced microglia activation

**Figure 6:**
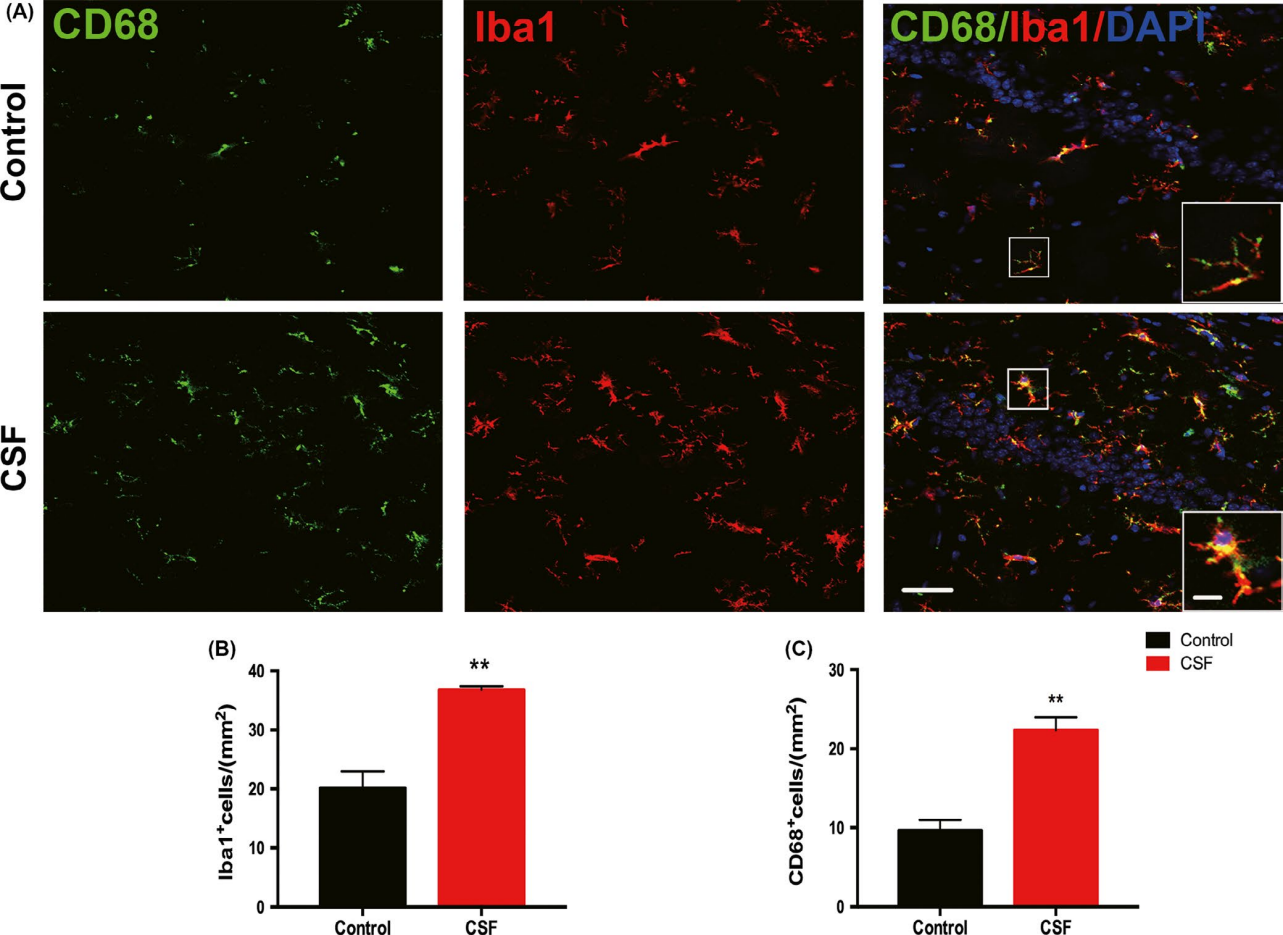
Activation of microglia in the hippocampus was induced by CSF. A, Representative confocal images labeled with Iba1 (red) and CD68 (green) in the hippocampus of CSF and control mice. Scale bar = 40 μm. Local enlarged images were presented in the white squares. Scale bar = 10 μm. B‐C, Statistical analysis of Iba1^+^ cells and CD68^+^ cells was shown in the histograms. n = 5 per group, **P < .01

Microglia activation with enhanced release of inflammatory mediators is known to exist in AD.^43^ Here, we investigated whether microglia activation also participated in the mechanism of CSF‐induced cognitive disorders. We doubled‐labeled microglia in brain slices by immunostaining for the general microglia marker Iba1 and the phagocytosis marker CD68 which was expressed in activated microglia (Figure 6A). Data showed that CSF could significantly increase the number of Iba1^+^ cells and CD68^+^ cells in the hippocampus region compared with the control (Figure 6B,C). While in the cortex, Iba1^+^ cells and CD68^+^ cells also tended to be higher in CSF mice, but the differences between the two groups were not statistically significant (Figure S1A‐C).

**Figure 7:**
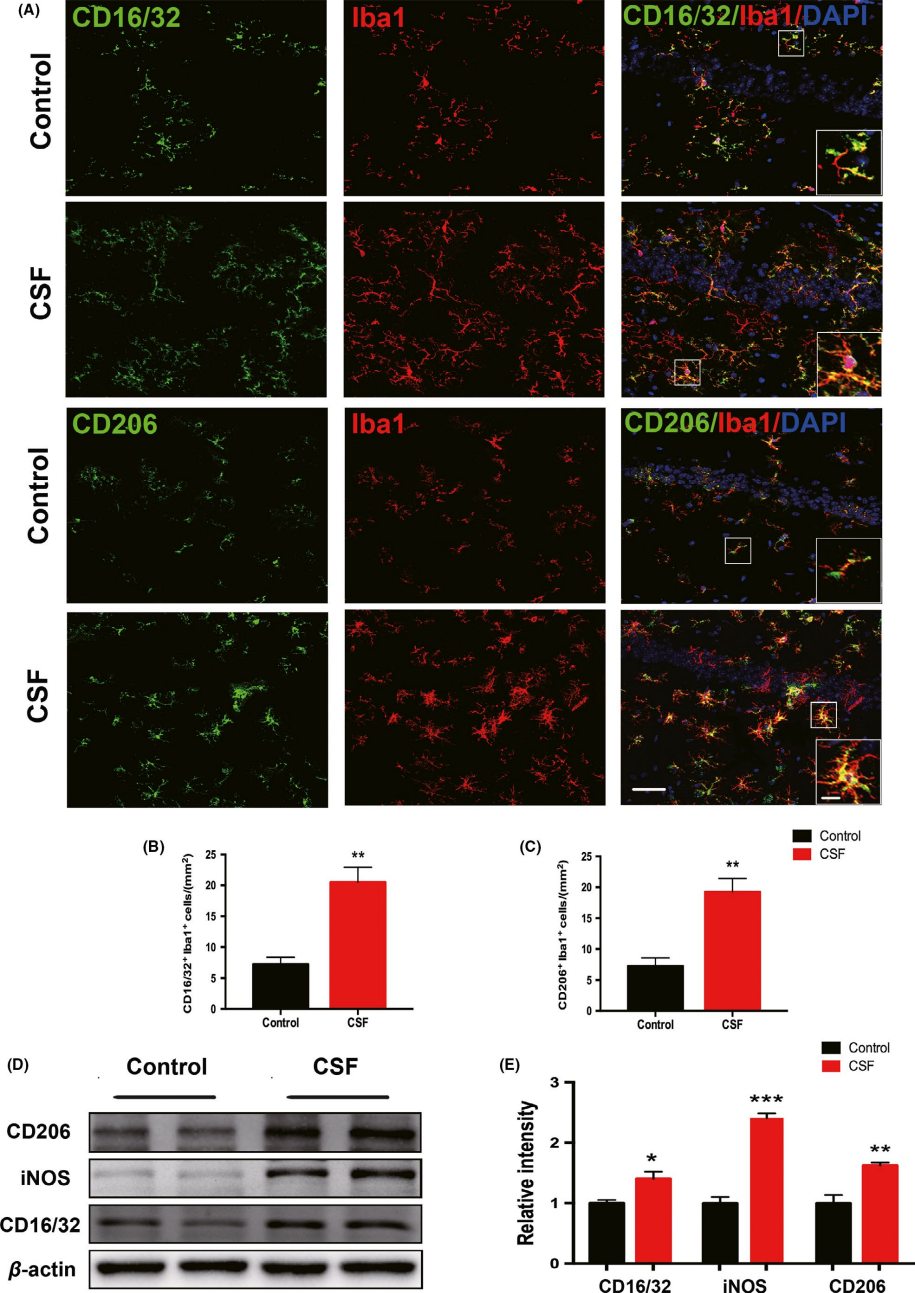
M1 and M2 polarized microglia in the hippocampus were activated after CSF. A, The M1 phenotype of microglia was detected by double staining for CD16/32 (green) and Iba1 (red), while the M2 phenotype was detected by double staining for CD206 (green) and Iba1 (red) in the hippocampus of CSF and control mice. Scale bar = 40 μm. Local enlarged images were presented in the white squares. Scale bar = 10 μm. B‐C, Quantitative analysis of Iba1^+^CD16/32^+^ double‐positive cells and Iba1^+^CD206^+^ double‐positive cells was shown in the histogram. n = 5 per group, **P < .01. D, Representative Western blots of CD16/32, iNOS, and CD206 in the hippocampus of CSF and control mice. E, Statistical analysis of Western blots of CD16/32, iNOS, and CD206 in the CSF and control groups. Protein expression levels were normalized to the level of β‐actin. n = 5 per group, *P < .05, **P < .01, ***P < .001

Activated microglia exists along a continuum of two functional states of polarization, namely the M1‐type (proinflammatory activation) and the M2‐type (antiinflammatory activation).^44^ Microglia with M1 phenotype are characterized by upregulation of CD16/32 and inducible nitric oxide synthase (iNOS), while microglia with M2 phenotype display overexpression of CD206. To explore whether CSF would affect the activated phenotypes of microglia, we performed double staining for Iba1 with either CD16/32 or CD206 (Figure 7A). The immunostaining results showed that the numbers of CD16/32^+^ Iba1^+^ cells and CD206^+^ Iba1^+^ cells were both increased in the hippocampus of CSF mice compared with control mice (Figure 7B,C). The Western blot for CD16/32, iNOS, and CD206 further confirmed that CSF could activate the microglia with increases in both M1‐and M2‐type markers in the hippocampus (Figure 7D,E). In cortex, there also existed elevated CD16/32 expression in CSF mice, but the CD206 level appeared consistent in two groups (Figure S1D,E).

## 4 DISCUSSION

In the present study, we found that CSF could induce pathogenic processes similar to those of early‐stage AD. In addition to impaired cognitive function, which was evident by behavior tests, we found intracellular Aβ accumulation after 2‐month CSF. Aβ, generated by cleavages of its precursor protein APP by β‐ and γ‐secretases, could be distributed in both intracellular and extracellular compartments in normal conditions.^45^, ^46^ In pathological conditions, the imbalance in the production, degradation, and clearance of Aβ was the key in the initiation of fibril and plaque formation and neurodegeneration. It was proposed that intracellular Aβ could be found in early‐stage but not late‐stage AD seen in human postmorterm autopsies.^46^ In another study, 5xFAD transgenic mice begin to develop intracellular Aβ accumulation as early as 2 months after birth, then gradually develop substantial extracellular plaques in the following months.^47^ Moreover, intracellular Aβ could be found in neurons, in nonneuronal cells, as well as could be located on the cell membrane.^48^ Therefore, the intracellular accumulation of Aβ evident in our study indicated that chronic sleep insufficiency could induce similar pathological changes with early stage or presymptomatic stage of AD. A few studies have shown that enhanced intracellular p‐Tau and Aβ accumulation were present after sleep deprivation in AD transgenic mice.^28^ However, our study is the first to report that intracellular Aβ can be found in wild‐type mice subjected to CSF. This implies a link between long‐term sleep insufficiency and cognitive impairment, which is probably related to AD pathogenesis, even without the genetic susceptibility.

**Figure 8:**
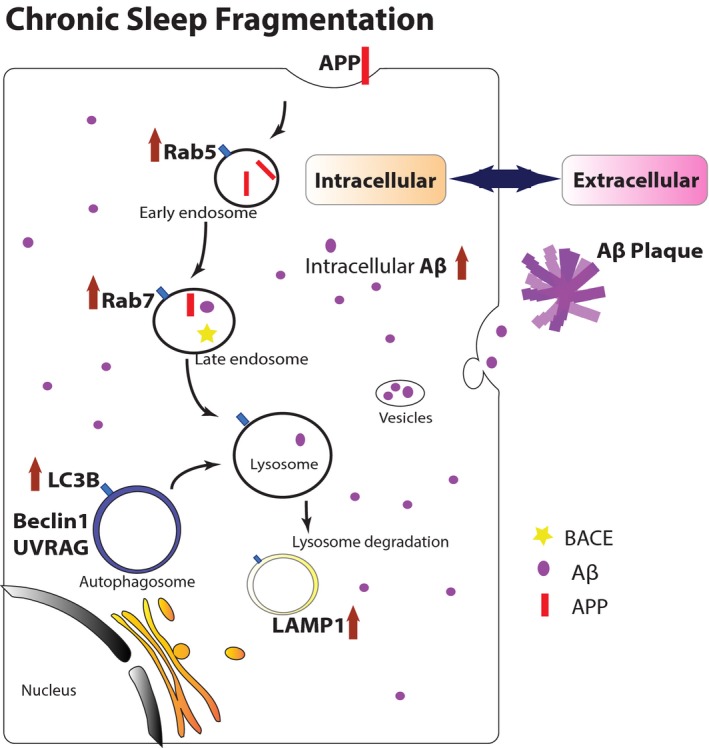
Schematic model of how CSF induced accumulation of endosomes, autophagosomes, and lysosomes, which led to increased intracellular Aβ accumulation. In normal conditions, the trafficking, catabolism, and elimination of APP are via the EAL pathway. First, APP is internalized into cells by endocytosis, then sorted in the early endosomes, and delivered to the late endosomes. APP can be processed by APP secretases to form Aβ in this stage. Then, the late endosomes fuse with either lysosomes or autophagosomes for lysosomal degradation. The balance of formation and clearance of Aβ is maintained by normal EAL pathway. After 2‐mo CSF, the EAL pathway is dysregulated and autophagic flux flow is impaired, leading to accumulation of abnormal vesicles in cells. It is evident by increased expression of Rab5 (early endosome marker), Rab7 (late endosome marker), Lamp1 (lysosome marker), as well as LC3B (autophagosome marker), and autophagy‐positive regulatory factors Beclin 1 and UVRAG. The accelerated APP processing in EAL pathway results in imbalance of formation and clearance of intracellular Aβ. In pathogenic process of Alzheimer's disease, overloaded intracellular Aβ can be released to extracellular space, potentially forming Aβ plaques eventually via oligomer polyermerization

In recent years, researchers in the AD field started to pay attention to the processes of trafficking, catabolism, and degradation of APP, which were together referred to as the EAL pathway in AD pathogenesis. In the EAL pathway, APP is processed and metabolized through the endocytosis of endosomes, the turnover of autophagosomes, and then degradation in lysosomes.^19^ Dysregulation of the EAL pathway was found in the different pathologic stages in AD transgenic mice, which was characterized by the accumulation of enlarged vacuoles containing APP and its metabolite Aβ.^20^ We previously published the evidence for the increased expression levels of endosome, autophagosome, and lysosome markers in APP/PS1 mice.^20^ In the current study, we found a consistent trend in the alteration of those markers after CSF by immunofluorescence staining and Western blot, which was shown schematically (Figure 8). Lysosomes are the primary cellular location to degrade varieties of biomacromolecules such as proteins and nucleic acids by fused with either endosomes or autophagosomes.^49^, ^50^ Using electron microscopy, we could clearly show the increase in the number and enlargement of intracellular lysosomes in the cortex of mice after CSF, which suggested a possible compensatory stage in order to remove excess intracellular Aβ.

In addition to the intracellular disordered degradation of Aβ, extracellular neuroinflammatory environment also participates in the pathogenesis of neurodegeneration. This process can affect glial reactions, oxidative stress, and deposition of Aβ.^27^, ^51^, ^52^ Aβ can also rapidly activate microglia and astrocytes, but nevertheless, sustained microglial activation and unresolved inflammation in the brain are harmful to neurons and synapses and even cause subsequent deterioration of brain structure and function. Briefly, neuroinflammation could accelerate the deposition of Aβ as well as other toxic materials, and vice versa.^53^, ^54^ It was reported highly significant, 2‐ to 5‐fold elevation in microglia number and area within Aβ deposits compared with neighboring regions in transgenic mouse models of AD.^55^ The density of activated microglia correlates with the severity of the inflammatory response.^56^ In this study, we found that activation of microglia after CSF predominately occurs in the hippocampus of mice. It can be interpreted that the hippocampal region is more vulnerable to neuroinflammation than cortex region after 2‐month sleep deficiency. Specifically, the phagocytosis marker CD68, which was expressed on activated microglia, was observed to be upregulated in CSF mice. Beyond that, two phenotypes of activated microglia, the M1 type characterized by CD16/32, iNOS and the M2 type characterized by CD206, were both found to be overexpressed to jointly generate a neuroinflammatory environment after CSF.

In current study, we used sleep fragmentation model for better mimicking sleep conditions of patients who are suffering from obstructive sleep apnea (OSA), periodic limb movements in sleep (PLMS), sleep maintenance insomnia, and other conditions inducing sleep disturbance.^30^ It was reported that the CSF model could effectively increase the wake time and reduce rapid eye movement (REM) sleep in mice during the light period, without effects on total sleep and wake times (24 hours) by EEG measurements.^30^, ^57^ Those results suggested that the CSF mice would adapt to reconstructed sleep patterns combining disrupted sleep period and mildly increased sleepiness during wake period. It was possible that circadian disruption or chronic stress was present in the mice of CSF group. As in real practice, individuals with sleep disturbance in the evening also experience a mild rebound of sleep‐time and inevitable stress in the daytime. Here, we mainly focused on whether the CSF could accelerate the development of cognitive disruption and early stage of AD. In a human study, it has been shown that only one night of sleep deprivation could significantly increase Aβ burden in the hippocampus and thalamus.^58^, ^59^ Aβ turnover in the brain follows circadian rhythms and is maintained in a dynamic status.^60^

For the first time, our study provided new evidence that CSF could induce a similar pathogenesis with early‐stage AD, including aggravated intracellular Aβ accumulation, dysfunction of EAL pathway, and activation of neuroinflammation in wild‐type mice. It suggested that, even if lack of genetic predisposition, Alzheimer‐like pathogenic changes could be possibly initiated in young adults after CSF. Patients suffering from OSA or sleep maintenance insomnia could potentially have higher risk to develop neurodegeneration pathogenesis than people with normal sleep habits. Consequently, early interventions for sleep disturbance would be potentially beneficial for prevention and slowing down development of neurodegenerative disease.

## CONFLICT OF INTEREST

The authors declare no conflict of interest.

## References

[1] Xie L , Kang H , Xu Q , et al. Sleep drives metabolite clearance from the adult brain. Science. 2013;6156:373‐377. 10.1126/science.1241224 PMC3880190 24136970

[2] Stickgold R , Walker M . Memory consolidation and reconsolidation: what is the role of sleep? Trends Neurosci. 2005;8:408‐415. 10.1016/j.tins.2005.06.004 15979164

[3] Kahn‐Greene ET , Killgore DB , Kamimori GH , Balkin TJ , Killgore WD . The effects of sleep deprivation on symptoms of psychopathology in healthy adults. Sleep Med. 2007;3:215‐221. 10.1016/j.sleep.2006.08.007 17368979

[4] Rosen IM , Gimotty PA , Shea JA , Bellini LM . Evolution of sleep quantity, sleep deprivation, mood disturbances, empathy, and burnout among interns. Acad Med. 2006;1:82‐85. 10.1097/00001888-200601000-00020 16377826

[5] Bubu OM , Brannick M , Mortimer J , et al. Sleep, cognitive impairment, and Alzheimer's disease: a systematic review and meta‐analysis. Sleep. 2017;1:zsw032. 10.1093/sleep/zsw032 28364458

[6] Ruiz Fernández MD , Ortega ÁM . Evaluation of the perceived health of caregivers of patients in mild‐to‐moderate stage Alzheimer's disease. Perspect Psychiatr Care. 2018;55(1):87‐94. 29969153 10.1111/ppc.12306

[7] Malhotra RK . Neurodegenerative disorders and sleep. Sleep Med Clin. 2018;1:63‐70. 10.1016/j.jsmc.2017.09.006 29412984

[8] Bero AW , Yan P , Roh JH , et al. Neuronal activity regulates the regional vulnerability to amyloid‐beta deposition. Nat Neurosci. 2011;6:750‐756. 10.1038/nn.2801 PMC3102784 21532579

[9] Hardy J . The Alzheimer family of diseases: many etiologies, one pathogenesis? Proc Natl Acad Sci USA. 1997;6:2095‐2097. 10.1073/pnas.94.6.2095 PMC33655 9122152

[10] Scheuner D , Eckman C , Jensen M , et al. Secreted amyloid beta‐protein similar to that in the senile plaques of Alzheimer's disease is increased in vivo by the presenilin 1 and 2 and APP mutations linked to familial Alzheimer's disease. Nat Med. 1996;8:864‐870. 10.1038/nm0896-864 8705854

[11] Mawuenyega KG , Sigurdson W , Ovod V , et al. Decreased clearance of CNS beta‐amyloid in Alzheimer's disease. Science. 2010;6012:1774. 10.1126/science.1197623 PMC3073454 21148344

[12] Iliff JJ , Wang M , Liao Y , et al. A paravascular pathway facilitates CSF flow through the brain parenchyma and the clearance of interstitial solutes, including amyloid beta. Sci Transl Med. 2012;147:147ra111. 10.1126/scitranslmed.3003748 PMC3551275 22896675

[13] Iliff JJ , Lee H , Yu M , et al. Brain‐wide pathway for waste clearance captured by contrast‐enhanced MRI. J Clin Invest. 2013;3:1299‐1309. 10.1172/JCI67677 PMC3582150 23434588

[14] Achariyar TM , Li B , Peng W , et al. Glymphatic distribution of CSF‐derived apoE into brain is isoform specific and suppressed during sleep deprivation. Mol Neurodegener. 2016;1:74. 10.1186/s13024-016-0138-8 PMC5146863 27931262

[15] Peng W , Achariyar TM , Li B , et al. Suppression of glymphatic fluid transport in a mouse model of Alzheimer's disease. Neurobiol Dis. 2016;93:215‐225. 27234656 10.1016/j.nbd.2016.05.015 PMC4980916

[16] Zhang R , Liu Y , Chen Y , et al. Aquaporin 4 deletion exacerbates brain impairments in a mouse model of chronic sleep disruption. CNS Neurosci Ther. 2019. 10.1111/cns.13194 PMC6978250 31364823

[17] Lista S , O'Bryant SE , Blennow K , et al. Biomarkers in sporadic and familial Alzheimer's disease. J Alzheimer's Dis: JAD. 2015;2:291‐317. 10.3233/JAD-143006 26401553

[18] Pan X‐D , Wei Z , Chen X‐C , et al. Amyloid β protein aggravates neuronal senescence and cognitive deficits in 5XFAD mouse model of Alzheimer's disease. Chin Med J. 2016;15:1835‐1844. 10.4103/0366-6999.186646 PMC4976573 27453234

[19] Nixon RA . Autophagy, amyloidogenesis and Alzheimer disease. J Cell Sci. 2007;23:4081‐4091. 10.1242/jcs.019265 18032783

[20] Ba L , Chen XH , Chen YL , et al. Distinct Rab7‐related endosomal‐autophagic‐lysosomal dysregulation observed in cortex and hippocampus in APPswe/PSEN1dE9 mouse model of Alzheimer's disease. Chin Med J (Engl). 2017;24:2941‐2950. 10.4103/0366-6999.220311 PMC5742922 29237927

[21] Yu WH , Cuervo AM , Kumar A , et al. Macroautophagy–a novel Beta‐amyloid peptide‐generating pathway activated in Alzheimer's disease. J Cell Biol. 2005;1:87‐98. 10.1083/jcb.200505082 PMC2171227 16203860

[22] Rubio‐Araiz A , Finucane OM , Keogh S , Lynch MA . Anti‐TLR2 antibody triggers oxidative phosphorylation in microglia and increases phagocytosis of beta‐amyloid. J Neuroinflammation. 2018;1:247. 10.1186/s12974-018-1281-7 PMC6119264 30170611

[23] Streit WJ , Braak H , Del Tredici K , et al. Microglial activation occurs late during preclinical Alzheimer's disease. Glia. 2018;12:2550‐2562. 10.1002/glia.23510 30417428

[24] Feng J , Wang JX , Du YH , et al. Dihydromyricetin inhibits microglial activation and neuroinflammation by suppressing NLRP3 inflammasome activation in APP/PS1 transgenic mice. CNS Neurosci Ther. 2018;12:1207‐1218. 10.1111/cns.12983 PMC6282966 29869390

[25] McGeer EG , McGeer PL , Lovell MA . Neuroinflammation in Alzheimer's disease and mild cognitive impairment: a field in its infancy. J Alzheimers Dis. 2010;1:355‐361. 10.3233/JAD-2010-1219 20061650

[26] Priller J , Krabbe G , Halle A , et al. Functional impairment of microglia coincides with beta‐amyloid deposition in mice with Alzheimer‐like pathology. PLoS ONE. 2013;4:e60921. 10.1371/journal.pone.0060921 PMC3620049 23577177

[27] Heppner FL , Ransohoff RM , Becher B . Immune attack: the role of inflammation in Alzheimer disease. Nat Rev Neurosci. 2015;6:358‐372. 10.1038/nrn3880 25991443

[28] Qiu H , Zhong R , Liu H , Zhang F , Li S , Le W . Chronic sleep deprivation exacerbates learning‐memory disability and Alzheimer's disease‐like pathologies in AβPPswe/PS1ΔE9 mice. J Alzheimers Dis. 2016;3:669‐685. 10.3233/JAD-150774 26757041

[29] Sinton CM , Kovakkattu D , Friese RS . Validation of a novel method to interrupt sleep in the mouse. J Neurosci Methods. 2009;1:71‐78. 10.1016/j.jneumeth.2009.07.026 19646474

[30] Li Y , Panossian LA , Zhang J , et al. Effects of chronic sleep fragmentation on wake‐active neurons and the hypercapnic arousal response. Sleep. 2014;1:51‐64. 10.5665/sleep.3306 PMC3902866 24470695

[31] D'Hooge R , De Deyn PP . Applications of the Morris water maze in the study of learning and memory. Brain Res Brain Res Rev. 2001;1:60‐90. 10.1016/s0165-0173(01)00067-4 11516773

[32] Vorhees CV , Williams MT . Morris water maze: procedures for assessing spatial and related forms of learning and memory. Nat Protoc. 2006;2:848‐858. 10.1038/nprot.2006.116 PMC2895266 17406317

[33] Ennaceur A , Delacour J . A new one‐trial test for neurobiological studies of memory in rats. 1: behavioral data. Behav Brain Res. 1988;1:47‐59. 10.1016/0166-4328(88)90157-x 3228475

[34] Tartar JL , Ward CP , Cordeira JW , et al. Experimental sleep fragmentation and sleep deprivation in rats increases exploration in an open field test of anxiety while increasing plasma corticosterone levels. Behav Brain Res. 2009;2:450‐453. 10.1016/j.bbr.2008.08.035 PMC2632847 18805441

[35] Han X , Wu H , Yin P , et al. Electroacupuncture restores hippocampal synaptic plasticity via modulation of 5‐HT receptors in a rat model of depression. Brain Res Bull. 2018;139:256‐262. 29524471 10.1016/j.brainresbull.2018.03.004

[36] Szentes N , Tekus V , Mohos V , Borbély É , Helyes Z . Exploratory and locomotor activity, learning and memory functions in somatostatin receptor subtype 4 gene‐deficient mice in relation to aging and sex. Geroscience. 2019. 10.1007/s11357-019-00059-1 PMC6885027 30903571

[37] Porsolt RD , Bertin A , Blavet N , Deniel M , Jalfre M . Immobility induced by forced swimming in rats: effects of agents which modify central catecholamine and serotonin activity. Eur J Pharmacol. 1979;2:201‐210. 10.1016/0014-2999(79)90366-2 488159

[38] Kandalepas PC , Sadleir KR , Eimer WA , Zhao J , Nicholson DA , Vassar R . The Alzheimer's beta‐secretase BACE1 localizes to normal presynaptic terminals and to dystrophic presynaptic terminals surrounding amyloid plaques. Acta Neuropathol. 2013;3:329‐352. 10.1007/s00401-013-1152-3 PMC3753469 23820808

[39] Song MS , Baker GB , Todd KG , Kar S . Inhibition of beta‐amyloid1‐42 internalization attenuates neuronal death by stabilizing the endosomal‐lysosomal system in rat cortical cultured neurons. Neuroscience. 2011;178:181‐188. 21262324 10.1016/j.neuroscience.2010.12.055

[40] Cataldo AM , Peterhoff CM , Troncoso JC , Gomez‐Isla T , Hyman BT , Nixon RA . Endocytic pathway abnormalities precede amyloid beta deposition in sporadic Alzheimer's disease and Down syndrome: differential effects of APOE genotype and presenilin mutations. Am J Pathol. 2000;1:277‐286. 10.1016/s0002-9440(10)64538-5 PMC1850219 10880397

[41] Grbovic OM , Mathews PM , Jiang Y , et al. Rab5‐stimulated up‐regulation of the endocytic pathway increases intracellular beta‐cleaved amyloid precursor protein carboxyl‐terminal fragment levels and Abeta production. J Biol Chem. 2003;33:31261‐31268. 10.1074/jbc.M304122200 12761223

[42] Kim KH , Lee MS . Autophagy–a key player in cellular and body metabolism. Nat Rev Endocrinol. 2014;6:322‐337. 10.1038/nrendo.2014.35 24663220

[43] Sarlus H , Heneka MT . Microglia in Alzheimer's disease. J Clin Invest. 2017;9:3240‐3249. 10.1172/JCI90606 PMC5669553 28862638

[44] Hu X , Li P , Guo Y , et al. Microglia/macrophage polarization dynamics reveal novel mechanism of injury expansion after focal cerebral ischemia. Stroke. 2012;11:3063‐3070. 10.1161/STROKEAHA.112.659656 22933588

[45] Bali J , Gheinani AH , Zurbriggen S , et al. Role of genes linked to sporadic Alzheimer's disease risk in the production of beta‐amyloid peptides. Proc Natl Acad Sci USA. 2012;38:15307‐15311. 10.1073/pnas.1201632109 PMC3458335 22949636

[46] Selkoe DJ . Alzheimer's disease results from the cerebral accumulation and cytotoxicity of amyloid beta‐protein. J Alzheimers Dis. 2001;1:75‐80. 10.3233/jad-2001-3111 12214075

[47] Shen H , Pan X‐D , Zhang J , et al. Endoplasmic reticulum stress induces the early appearance of pro‐apoptotic and anti‐apoptotic proteins in neurons of five familial Alzheimerʼs disease mice. Chin Med J. 2016;23:2845‐2852. 10.4103/0366-6999.194643 PMC5146794 27901000

[48] Hartmann T , Bieger SC , Bruhl B , et al. Distinct sites of intracellular production for Alzheimer's disease A beta40/42 amyloid peptides. Nat Med. 1997;9:1016‐1020. 10.1038/nm0997-1016 9288729

[49] Mony VK , Benjamin S , O'Rourke EJ . A lysosome‐centered view of nutrient homeostasis. Autophagy. 2016;4:619‐631. 10.1080/15548627.2016.1147671 PMC4836021 27050453

[50] Huang J , Wang X , Zhu Y , et al. Exercise activates lysosomal function in the brain through AMPK‐SIRT1‐TFEB pathway. CNS Neurosci Ther. 2019;6:796‐807. 10.1111/cns.13114 PMC6515701 30864262

[51] Aktas O , Ullrich O , Infante‐Duarte C , Nitsch R , Zipp F . Neuronal damage in brain inflammation. Arch Neurol. 2007;2:185‐189. 10.1001/archneur.64.2.185 17296833

[52] Agrawal SM , Yong VW . The many faces of EMMPRIN—roles in neuroinflammation. Biochim Biophys Acta (BBA) ‐ Mol Basis Dis. 2011;2:213‐219. 10.1016/j.bbadis.2010.07.018 20674741

[53] Koenigsknecht J , Landreth G . Microglial phagocytosis of fibrillar beta‐amyloid through a beta1 integrin‐dependent mechanism. J Neurosci. 2004;44:9838‐9846. 10.1523/JNEUROSCI.2557-04.2004 PMC6730228 15525768

[54] Steardo L Jr , Bronzuoli MR , Iacomino A , Esposito G , Steardo L , Scuderi C . Does neuroinflammation turn on the flame in Alzheimer's disease? Focus on astrocytes. Front Neurosci. 2015;9:259. 26283900 10.3389/fnins.2015.00259 PMC4518161

[55] Frautschy SA , Yang F , Irrizarry M , et al. Microglial response to amyloid plaques in APPsw transgenic mice. Am J Pathol. 1998;152(1):307‐317. 9422548 PMC1858113

[56] Carpenter AF , Carpenter PW , Markesbery WR . Morphometric analysis of microglia in Alzheimer's disease. J Neuropathol Exp Neurol. 1993;52(6):601‐608. 8229079 10.1097/00005072-199311000-00007

[57] Ramesh V , Nair D , Zhang SX , et al. Disrupted sleep without sleep curtailment induces sleepiness and cognitive dysfunction via the tumor necrosis factor‐alpha pathway. J Neuroinflammation. 2012;9:91. 22578011 10.1186/1742-2094-9-91 PMC3411474

[58] Shokri‐Kojori E , Wang GJ , Wiers CE , et al. beta‐Amyloid accumulation in the human brain after one night of sleep deprivation. Proc Natl Acad Sci USA. 2018;17:4483‐4488. 10.1073/pnas.1721694115 PMC5924922 29632177

[59] Rezai‐Zadeh K , Gate D , Town T . CNS infiltration of peripheral immune cells: D‐day for neurodegenerative disease? J Neuroimmune Pharmacol. 2009;4:462‐475. 19669892 10.1007/s11481-009-9166-2 PMC2773117

[60] Kang JE , Lim MM , Bateman RJ , et al. Amyloid‐beta dynamics are regulated by orexin and the sleep‐wake cycle. Science. 2009;5955:1005‐1007. 10.1126/science.1180962 PMC2789838 19779148

