# Supplementary figures and images for: Chronic sleep fragmentation shares similar pathogenesis with neurodegenerative diseases: Endosome‐autophagosome‐lysosome pathway dysfunction and microglia‐mediated neuroinflammation
Source: CNS Neurosci Ther. 2019 Sep 24;26(2):215–27. doi: 10.1111/cns.13218 (PMC6978272; doi:10.1111/cns.13218)

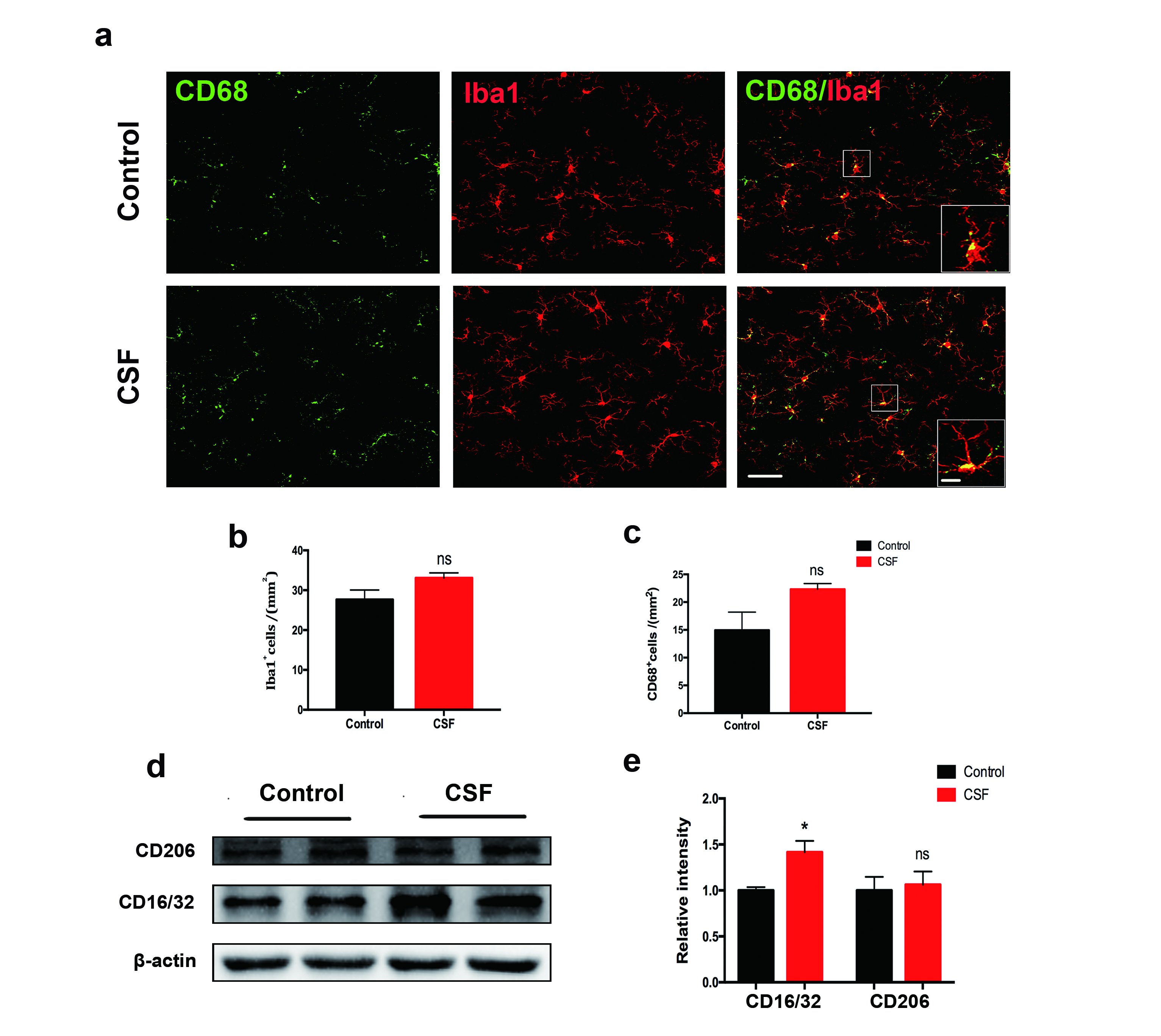

Supplement: Supplementary file 1 [file CNS-26-215-s001.tif]
