# Supplementary material for: Chronic sleep fragmentation shares similar pathogenesis with neurodegenerative diseases: Endosome‐autophagosome‐lysosome pathway dysfunction and microglia‐mediated neuroinflammation
Source: CNS Neurosci Ther. 2019 Sep 24;26(2):215–27. doi: 10.1111/cns.13218 (PMC6978272; doi:10.1111/cns.13218)
Supplement: Supplementary file 2 [file CNS-26-215-s002.docx]

**Supplementary Materials**

**Fig.S1 Microglia activation was mild in the cortex after chronic sleep fragmentation. (a)** Representative confocal images labeled with Iba1 (red) and CD68 (green) in the cortex of CSF and control mice. Scale bar=40 μm. Local enlarged images were presented in the white squares. Scale bar=10 μm**. (b-c)** Statistical analysis of Iba1+ and CD68+ cells was shown in the histograms. n = 5 per group. n.s. indicates no significant changes between different groups. **(d)** Representative western blots of CD16/32 and CD206 in the cortex of CSF and control groups. **(e)** Statistical analysis of western blots signals of CD16/32 and CD206 in the CSF and control groups .The protein expression levels were normalized to the level of β-actin. n = 5 per group, n.s. indicates no significant changes between different groups, *P< 0.05.
